# Non-nutritive sweeteners improve growth, reduce diarrhea, and modulate intestinal and systemic metabolism in weaned pigs

**DOI:** 10.1093/jas/skag005

**Published:** 2026-01-14

**Authors:** Mariah R Jansen, Charlotte Ludorf, Riley E Barber, Veronica I Polniak, Andrea M Luttman, Dale W Rozeboom, Kwangwook Kim

**Affiliations:** Department of Animal Science, Michigan State University, East Lansing, MI 48824; Department of Animal Science, Michigan State University, East Lansing, MI 48824; Department of Animal Science, Michigan State University, East Lansing, MI 48824; Hord Family Farms, Bucyrus, OH 44820; Department of Animal Science, Michigan State University, East Lansing, MI 48824; Department of Animal Science, Michigan State University, East Lansing, MI 48824; Department of Animal Science, Michigan State University, East Lansing, MI 48824

**Keywords:** diarrhea, growth, gut health, immune response, non-nutritive sweeteners, weaned pigs

## Abstract

Early weaning improves swine productivity but induces stress that impairs growth, compromises intestinal health, and increases diarrhea. A total of 288 weaned pigs (21 ± 1 d; PIC 800× Yorkshire; initial body weight (BW) 6.21 ± 0.45 kg) were used in a randomized complete block design, with initial BW as the blocking factor and pen as the experimental unit (48 pens; 6 pig/pen). Pigs were assigned to one of four dietary treatments: a nursery basal diet (control; CON), CON supplemented with 150 mg/kg sucralose (SCL). CON supplemented with 30 mg/kg neotame (NEO) or CON supplemented with 50 mg/kg carbadox (CBX). This study investigated the effects of dietary SCL or NEO supplementation on growth performance, diarrhea incidence, immune responses, intestinal development, and serum metabolites in weaned pigs. Pigs supplemented with SCL tended to increase (*P *= 0.093) average daily gain (ADG) from day 0 to 7 and increased (*P *< 0.05) average daily feed intake (ADFI) during phase 1 compared with CON. Pigs supplemented with NEO tended to increase (*P *= 0.083, *P *= 0.090) BW on days 7 and 14 and ADG (*P *= 0.053), and increased ADFI (*P *< 0.05) during phase 1 compared with CON. Both NEO (*P *< 0.05) and SCL (*P *< 0.10) reduced diarrhea frequency during phase 1 and across the experimental period compared with CON. SCL improved villus height-to-crypt depth ratio (*P *< 0.05), and reduced crypt depth (*P *< 0.05) compared with CON on day 14. SCL downregulated (*P *< 0.05) tight junction protein 1 (TJP1) expression compared with CBX on day 28. Untargeted serum metabolomics revealed that both SCL and NEO altered amino acid, nucleoside, antioxidant, and lipid metabolic pathways relative to CON. On day 14, SCL altered β-alanine and glutathione metabolism, whereas NEO modulated amino acid-derived metabolites. By day 28, SCL modulated purine, D-amino acid, and ether lipid metabolism, while NEO was associated with taurine and hypotaurine metabolism. These findings indicate that SCL and NEO improve growth performance and reduce post-weaning diarrhea, with SCL additionally enhancing intestinal structure and barrier-related markers, while NEO may act through palatability enhancements or microbiota-associated metabolic pathways.

## Teaser

Non-nutritive sweeteners were supplemented to weaned pigs to evaluate their effects on growth, gut development and function, immune responses, and systemic metabolism.

## Introduction

In commercial swine production, weaning between 2 and 4 wk of age is commonly practiced to improve production efficiency, optimize sow productivity, and increase profitability (Moeser, Pohl, and Rajput, 2017). However, early weaning imposes substantial physiological stress due to abrupt maternal separation, dietary transition, and environmental changes (Tang et al., 2022). These combined stressors, termed post-weaning stress, can negatively affect feed intake, growth performance, intestinal integrity, and immune responses, frequently leading to diarrhea and heightened inflammation (Upadhaya and Kim, 2021). Consequently, mortality rates can reach up to 17.6% within the first 4 wk post-weaning, significantly impacting overall productivity (National Pork Board and Meta Farms, 2023). Historically, the swine industry routinely uses in-feed antibiotics at growth-promoting doses to mitigate post-weaning stress by enhancing growth, feed intake, and supporting intestinal health (Keegan et al., 2005). However, growing regulatory limitations on antibiotic usage in livestock diets have stimulated interest in identifying effective nutritional alternatives during this critical post-weaning period.

Several nutritional strategies have been explored, including low-protein diets, improved feed processing techniques, and the incorporation of functional feed additives (Lynegaard et al., 2021). Of these strategies, feed additives have garnered particular attention due to their potential to enhance gut development and function, modulate immune responses, and stimulate growth performance in newly weaned pigs. Commonly studied feed additives include direct-fed microbials (Su et al., 2022), prebiotics (Kiernan et al., 2023), organic acids (Xiang et al., 2021), and plant-derived compounds such as essential oils and extracts (Shao et al., 2023). Among these feed additives, non-nutritive sweeteners (NNS) were selected for investigation in the present study due to their potential to enhance feed intake and growth performance in weaned pigs, particularly during the stress-sensitive post-weaning period.

NNS, also known as artificial or high-intensity sweeteners, are compounds possessing minimal caloric value and sweetness intensities ranging from 10 to 1,000 times that of sucrose (Chen et al., 2020). These sweeteners initially gained prominence in human nutrition during the 1960s as a strategy to lower calorie intake amid growing concerns related to obesity, diabetes, and cardiovascular disease (Jain et al., 2017). Various NNS have been approved by regulatory agencies such as the FDA and the European Commission, including advantame, aspartame, acesulfame-K, cyclamate, neohesperidin dihydrochalcone, neotame, saccharin, sucralose, steviosides, and thaumatin (European Parliament, 2006; FDA, 2024). These compounds were subsequently introduced into livestock diets to enhance feed palatability and promote intake during periods of production-related stress. In swine, especially during early weaning, dietary NNS supplementation has demonstrated improvements in feed preference (Chen et al., 2020; Zhang et al., 2020), as well as increased feed intake and growth performance (Lee et al., 2019; Xiong et al., 2022).

Furthermore, several studies have reported beneficial effects of NNS on reducing post-weaning diarrhea incidence (Sterk et al., 2008; Liu et al., 2022), enhancing intestinal development, and positively influencing immune responses (Zhang et al., 2020; Xiong et al., 2022). Specifically, neotame and sucralose have shown potential in improving average daily feed intake (ADFI) and average daily gain (ADG) in weaned pigs. Neotame supplementation resulted in linear and quadratic improvements in pig performance, with optimal dietary inclusion levels ranging from 20.7 to 21.7 mg/kg (Lee et al., 2019; Xiong et al., 2022). Similarly, sucralose inclusion at 150 mg/kg consistently achieved the highest improvements in ADFI and ADG, with optimal levels estimated between 137 and 150 mg/kg depending on the duration of supplementation (Zhang et al., 2020). Moreover, sucralose supplementation has been linked with increased expression of intestinal nutrient transporters, notably sodium-glucose cotransporter (*SGLT1*) and sweet taste receptors T1R2 and T1R3 (Daly et al., 2021).

Despite these findings, limited research has examined the broader physiological impacts of NNS supplementation, such as effects on immune responses, serum biochemical parameters, intestinal morphology, and diarrhea incidence in weaned pigs. Based on prior evidence, we hypothesized that dietary supplementation of sucralose or neotame would enhance growth performance, reduce diarrhea incidence, modulate immune function and systemic metabolism, and support intestinal development comparable to in-feed antibiotics, such as carbadox. Therefore, this study aimed to evaluate the effects of dietary supplementation with sucralose or neotame on growth performance, diarrhea incidence, immune responses, serum metabolites, and intestinal development in weaned pigs.

## Materials and Methods

The experimental protocol was reviewed and approved by the Michigan State University Institutional Animal Care and Use Committee (MSU IACUC; Protocol #PROTO202300055).

### Animals, housing, experimental design, and diet

The experiment was conducted at the MSU Swine Teaching and Research Center. A total of 288 weaned pigs (21 ± 1 d; PIC 800× Yorkshire; initial body weight (BW): 6.21 ± 0.45 kg), comprising an equal number of barrows and gilts and sourced from multiparous sows, were obtained from two consecutive 4-wk studies; data from both studies were pooled for analysis. At weaning, pigs were randomly allotted to one of four dietary treatments in a randomized complete block design, using initial BW (heavy to light) as a blocking factor and pen as the experimental unit across 12 blocks. Pigs were housed in one of the 48 pens (1.22 × 1.83 m per pen), with six pigs per pen, in a mechanically ventilated nursery room for 28 d. Each pen was equipped with round-rod steel flooring, vertical-rod fiberglass fencing and gates, a single-sided two-hole feeder, and one nipple drinker. Pen-to-pen contamination among treatments was considered minimal because pens and alleyways were cleaned regularly.

The four dietary treatments were: 1) a nursery basal diet (control; CON); 2) CON supplemented with 150 mg/kg sucralose (SCL); 3) CON supplemented 30 mg/kg neotame (NEO); and 4) CON supplemented with 50 mg/kg carbadox (CBX). The in-feed antibiotic used in this study was carbadox (Phibro Animal Health Corporation, Teaneck, NJ), while the NNS were sucralose and neotame (Sigma-Aldrich Inc., St Louis, MO, USA). Diets did not include spray-dried plasma or zinc oxide at levels exceeding typical industry recommendation or standard practices. All diets were formulated to meet the nutritional requirements of nursery pigs (NRC, 2012) and were provided in mash form throughout the study (Table 1). A two-phase feeding program was employed, with phase 1 spanning the first 2 wk (14 d) and phase 2 covering the final 2 wk of the study (14 d). Each experimental diet was prepared by thoroughly mixing the specific additive with the appropriate amount of the basal control diet. Diets were mixed onsite at the MSU Swine Teaching and Research Center using a 113 kg paddle ribbon mixer. The mixer was emptied and cleaned thoroughly between batches to minimize cross-contamination. Experimental diet and water were provided *ad libitum* throughout the duration of the study.

**Table 1.** Ingredient compositions of experimental diets**^1^**

Ingredient, %	Control, phase I	Control, phase II
**Corn**	44.41	57.27
**Dried whey**	15.00	10.00
**Soybean meal**	18.00	22.00
**Fish meal**	10.00	7.00
**Lactose**	6.00	–
**Soy protein concentrate**	3.00	–
**Soybean oil**	2.00	2.00
**Limestone**	0.56	0.70
**L-lysine·HCl**	0.21	0.23
**DL-methionine**	0.08	0.05
**L-threonine**	0.04	0.05
**Salt**	0.40	0.40
**Vit-mineral, Sow 6^2^**	0.30	0.30
**Total**	100.00	100.00
**Calculated energy and nutrient**		
** Metabolizable energy, kcal/kg**	3,463	3,429
** Net energy, kcal/kg**	2,601	2,575
** Crude protein, %**	22.27	20.80
** SID Arg,^3^ %**	1.23	1.15
** SID His,^3^ %**	0.49	0.47
** SID Ile,^3^ %**	0.83	0.76
** SID Leu,^3^ %**	1.62	1.55
** SID Lys,^3^ %**	1.35	1.23
** SID Met,^3^ %**	0.45	0.39
** SID Thr,^3^ %**	0.79	0.73
** SID Trp,^3^ %**	0.23	0.21
** SID Val,^3^ %**	0.91	0.84
** SID Met + Cys,^3^ %**	0.74	0.68
** SID Phe + Tye,^3^ %**	1.45	1.38
** Ca, %**	0.80	0.70
** Total P, %**	0.68	0.59
** Digestible P, %**	0.47	0.37

1 In each phase, three additional diets will be formulated by adding 150 mg/kg sucralose, 30 mg/kg of neotame, or 50 mg/kg or carbadox to the control diet, respectively.

2 Provides the following quantities of vitamins and micro minerals per kilogram of complete diet: Vitamin A as retinyl acetate, 11,136 IU; vitamin D3 as cholecalciferol, 2,208 IU; vitamin E as DL-alpha tocopheryl acetate, 66 IU; vitamin K as menadione dimethylprimidinol bisulfite, 1.42 mg; thiamin as thiamine mononitrate, 0.24 mg; riboflavin, 6.59 mg; pyridoxine as pyridoxine hydrochloride, 0.24 mg; vitamin B12, 0.03 mg; D-pantothenic acid as D-calcium pantothenate, 23.5 mg; niacin, 44.1 mg; folic acid, 1.59 mg; biotin, 0.44 mg; Cu, 20 mg as copper sulfate and copper chloride; Fe, 126 mg as ferrous sulfate; I, 1.26 mg as ethylenediamine dihydriodide; Mn, 60.2 mg as manganese sulfate; Se, 0.3 mg as sodium selenite and selenium yeast; and Zn, 125.1 mg as zinc sulfate.

3 SID, Standardized ileal digestibility, refers to the portion of an amino acid in a feed ingredient that is digested and absorbed by the animal and is corrected for basal endogenous amino acid losses.

### Clinical observations and sample collections

The procedures for this experiment were adapted from methods previously described by Kim et al. (2019). Pig BW and feed consumption were measured and recorded for each interval: from weaning day (day 0) to 7, day 7 to 14, day 14 to 21, and day 21 to 28 post-weaning. ADG, ADFI, and gain-to-feed ratio (G:F) were calculated based on these measurements. The overall mortality rate observed throughout the experiment was 0.694%, which did not differ among dietary groups and had no impact on other measurements.

Clinical observations, including diarrhea and alertness scores, were recorded twice daily throughout the study, beginning on day 0. Diarrhea scores were assigned by two independent evaluators using a scale of 1 to 5 (1 = normal feces, 2 = moist feces, 3 = mild diarrhea, 4 = severe diarrhea, 5 = watery diarrhea). The frequency of diarrhea was calculated as the percentage of pen days with a diarrhea score of 3 or greater. The alertness score for each pen was visually assessed using a 3-point scale (1 = normal, 2 = slightly depressed or listless, and 3 = severely depressed or recumbent). Before beginning the evaluations, both scoring systems were calibrated among evaluators through collaborative visual assessments. All pens consistently exhibited an alertness score of 1 throughout the study; therefore, data is not reported.

Whole blood samples were collected via jugular vein puncture from six pigs per treatment (one pig selected per every two pens, with pen considered the experimental unit) on days 0, 3, 7, and 14, and day 28, using Vacutainer Eclipse blood collection needles and Vacutainer serum blood collection tubes without ethylenediaminetetraacetic acid (EDTA) (Becton, Dickinson and Company, Franklin Lakes, NJ). Serum was obtained by centrifuging whole blood samples at 1,500 x g for 15 min at 20 °C and then transferred into 1.7 mL graduated microcentrifuge tubes using disposable transfer pipettes. Serum samples were promptly stored at −80 °C for further analysis.

At the conclusion of each feeding phase, 48 pigs (12 pigs per treatment) were randomly selected from pens within each treatment, with sex balanced, for euthanasia. Prior to euthanasia, pigs were anesthetized by intramuscular injection with 1 mL of an anesthetic mixture containing 50 mg ketamine and 50 mg xylazine in a 1:1 ratio to ensure deep sedation. Following anesthesia, euthanasia was performed via intracardiac injection of sodium pentobarbital at a dosage of 78 mg/kg BW consistent with AVMA guidelines for swine. The euthanasia procedure and dosage were discussed with the MSU South Farm veterinarian and reviewed and approved by the MSU IACUC, ensuring a humane and rapid death. Three segments, each approximately 4 cm in length, were collected from the duodenum, middle jejunum, and ileum (10 cm proximal to the ileocecal junction) of each euthanized pig and fixed in 10% formalin for intestinal morphology assessment. Separate 4 cm segments of the jejunum and ileum were used to collect mucosal samples. After gently rinsing the intestinal lumen with phosphate-buffered saline (PBS), the mucosal layer was carefully scraped using a glass slide, transferred to cryovials, and snap-frozen in liquid nitrogen for subsequent gene expression analysis by quantitative real-time PCR (qRT-PCR). Samples were then stored at –80 °C until analysis.

### Measurements of serum cytokine and acute phase proteins

Serum samples (*n* = 6) were analyzed for pro-inflammatory cytokines (tumor necrosis factor-α (TNF-α); R&D Systems Inc., Minneapolis, MN) and acute phase proteins (C-reactive protein and haptoglobin; Aviva Systems Biology Corp., San Diego, CA) using porcine-specific enzyme-linked immunosorbent assay (ELISA) kits. All serum samples, standards, and controls were analyzed in duplicate to ensure assay reliability. TNF-α was measured by adding samples, standards, and controls to a 96-well polystyrene microplate pre-coated with a monoclonal antibody specific to porcine TNF-α. Following a 2-h incubation, unbound substances were removed using a diluted wash buffer and a MultiWash + microplate washer (Molecular Devices, LLC., San Jose, CA). A horseradish peroxidase-conjugated monoclonal antibody was then added to bind the immobilized TNF-α. After a second 2-h incubation, the wells were washed again, and a substrate solution containing stabilized hydrogen peroxide and chromogen was added. The intensity of color development was proportional to the amount of TNF-α bound during the initial incubation. After a 30-min light-protected incubation, a stop solution (diluted hydrochloric acid) was added to terminate the reaction. Absorbance was measured at 450 nm with a correction wavelength of 540 nm using a plate reader (BioTek 800TS, BioTek Instruments, Inc., Winooski, VT). Haptoglobin and C-reactive protein were measured using similar ELISA procedures, with modifications to sample pre-treatment and incubation times as specified by the manufacturer. The intra-assay coefficients of variation (CV) for TNF-α, C-reactive protein, and haptoglobin were 6.9%, 5.28%, and 5.00%, respectively. The inter-assay CV for TNF-α, C-reactive protein, and haptoglobin were 9.2%, 7.01%, and 5.35%, respectively. Concentrations of TNF-α, C-reactive protein, and haptoglobin were calculated using a standard curve and expressed as picograms or nanograms per milliliter, depending on the analyte Samples with CV values exceeding 10% were reanalyzed to ensure data accuracy.

### Untargeted serum metabolomics analysis

The untargeted metabolomics analysis was performed on serum samples (*n* = 6) by the NIH West Coast Metabolomics Center using gas chromatography (Agilent 6890 gas chromatograph, Agilent, Santa Clara, CA, USA) coupled with time-of-flight mass spectrometry (GC/TOF–MS; Leco Pegasus IV time-of-flight mass spectrometer, Leco, St. Joseph, MI, USA), both controlled using Leco ChromaTOF software version 2.32. Metabolite extraction was performed following procedures described previously by Fiehn et al. (2008). Frozen serum samples (approximately 30 μL) were homogenized in a Retsch ball mill (Retsch, Newtown, PA, USA) for 30 s at 25 Hz. A prechilled (−20 °C) extraction solution (isopropanol/acetonitrile/water, 3:3:2, v/v/v; degassed with liquid nitrogen) was then added at 1 mL per 20 mg of sample. Samples were vortexed and shaken, followed by centrifugation at 12,800 × g for 2 min. The supernatant was collected, divided into two aliquots, and concentrated at room temperature for 4 h in a cold-trap vacuum concentrator (Labconco Centrivap, Kansas City, MO, USA). To remove complex lipids and waxes, residues were re-suspended in 500 µL of 50% aqueous acetonitrile and centrifuged at 12,800 × g for 2 min. The resulting supernatant was collected and concentrated again in the vacuum concentrator. Dried extracts were derivatized and mixed with internal retention index markers (fatty acid methyl esters, C8–C30) before injection for GC/TOF–MS analysis. All samples were analyzed in a single batch. Mass spectrometry data acquisition included calibration with FC43 (perfluorotributylamine) prior to each analytical sequence. Metabolite identification was based on two criteria: 1) retention index within ± 2,000 U (≈ ± 2 s retention time deviation), and 2) mass spectral similarity, with additional confidence criteria described in Fiehn et al. (2008).

### Intestinal morphology

Fixed tissue segments from the duodenum, jejunum, and ileum (*n* = 12) were trimmed into two 5 mm slices using a scalpel, placed into plastic cassettes, and soaked in 50% ethanol for further dehydration. Samples were embedded in paraffin, sectioned at 5 µm thickness, and stained with hematoxylin and eosin at the MSU Histology Laboratory. Stained slides were scanned at 20 X magnification using an Aperio VERSA imaging system (Leica Biosystems, Lincolnshire, IL) at the MSU Precision Health Program Tissue Analysis Core. Morphometric measurements were performed using ImageScope software (Leica Biosystems, Vista, CA). For each slide, 10 to 15 well-oriented and intact villi along with their associated crypts were selected to assess villus height, width, and area, as well as crypt depth.

### Intestinal barrier and innate immunity

Jejunal and ileal mucosa samples (*n* = 6) were collected for gene expression analysis using qRT-PCR. Total RNA was extracted using TRIzol reagent (Invitrogen, Thermo Fisher Scientific, Waltham, MA). For each sample, approximately 50 mg of mucosa tissue and 1 mL TRIzol reagent were placed into microcentrifuge tubes with three 2.4-mm stainless-steel beads and homogenized at 180 Hz for 2 min using the TissueLyser System II (QIAGEN, Hilden, Germany). After homogenization, 200 µL of chloroform (Macron Fine Chemicals, Center Valley, PA) was added to the TRIzol solution in a new tube. Tubes were vortexed for 15 s and incubated at 4 °C for 3 min. The samples were then centrifuged at 12,000 × g for 15 min at 4 °C. The upper aqueous phase (400 µL) was transferred to a new tube, and 400 µL of isopropanol (Fisher Chemical, Pittsburgh, PA) was added. Tubes were gently inverted 10 times and incubated at 4 °C for 10 min. Samples were then centrifuged again at 12,000 × g for 10 min at 4 °C. After removing the supernatant, the pellet was washed with 1 mL of 70% ethanol (–20 °C) and centrifuged at 7,500 × g for 5 min at 4 °C. The supernatant was again discarded, and pellets were air-dried under a fume hood for 10 min. After drying, RNA pellets were reconstituted in DEPC-treated water (Fisher BioReagents, Pittsburgh, PA) and vortexed. RNA quality and concentration were assessed using a NanoDrop One/One Microvolume UV-Vis Spectrophotometer (Thermo Fisher Scientific, Waltham, MA). RNA samples were diluted to 1000 ng/mL using DEPC-treated water for complementary DNA (cDNA) synthesis. cDNA was synthesized in 20 µL reactions using the High-Capacity cDNA Reverse Transcription Kit (Applied Biosystems; Thermo Fisher Scientific, Inc., Waltham, MA), following the manufacturer’s instructions. Following synthesis, cDNA was quantified, diluted to 100 µg/µL using nuclease-free water (Invitrogen, Thermo Fisher Scientific, Waltham, MA), and stored at –20 °C. qRT-PCR was performed using the QuantStudio 6 Pro system (Applied Biosystems, Thermo Fisher Scientific, Waltham, MA) to assess the expression of genes associated with intestinal barrier integrity, mucosal development, and innate immune responses. In jejunal mucosa, the expression of mucin 2 (*MUC2*), claudin 1 (*CLDN1*), occludin (*OCLN*), tumor necrosis factor-α (*TNF-*α*)*, sodium glucose cotransporter-1 (*SLC5A1*), glucagon-like peptide 2 receptor (*GLP2R*), and tight junction protein-1 (*TJP1*) was analyzed by qRT-PCR. In ileal mucosa, gene expression of *TNF-*α, claudin-1 *(CLDN1)*, interleukin-1a *(IL1*a*)*, interleukin-1β *(IL1β)*, interleukin-6 *(IL6)*, interleukin-7 *(IL7)*, interleukin-10 *(IL10)* was evaluated. Gene expression was normalized using ribosomal protein S18 (*RPS18*) as the reference gene for both jejunal and ileal samples. Relative gene expression was quantified using the TaqMan Fast Advanced Master Mix and TaqMan Gene Expression Assays (Applied Biosystems, Thermo Fisher Scientific, Waltham, MA). All TaqMan assays were validated prior to qRT-PCR analysis (Supplementary Table S1). Reactions were run in duplicate under manufacturer-recommended thermal conditions: an initial denaturation at 95 °C for 20 s, followed by 45 cycles of 95 °C for 1 s and 60 °C for 20 s. Melt curve analysis was performed using a continuous cycle of 95 °C for 1 s, 60 °C for 20 s, and a final step at 95 °C for 1  s. All qRT-PCR data were acquired using the QuantStudio 6 Pro system (Thermo Fisher Scientific, Waltham, MA) and analyzed via the Thermo Fisher Cloud platform. Cycle threshold (Ct) values were processed using the Thermo Fisher’s Relative Quantification application, applying the ^ΔΔ^ct method for normalization. The CON group was designated as the reference treatment, and all fold-change values for other treatments were calculated relative to CON. Statistical analyses were conducted on 2^-^ΔΔCt fold change values, and the results are presented in tables and figures as relative expression (2^-^ΔΔCt) reported as mean ± SEM.

### Statistical analysis

Data normality was assessed using a Shapiro-Wilk test, and outliers were identified using the UNIVARIATE procedure in SAS (SAS Institute, Inc., Cary, NC). All data were analyzed using analysis of variance (ANOVA) with the PROC MIXED procedure in SAS, following a randomized complete block design. Frequency of diarrhea data were analyzed using a Chi-squared test. Pen was considered the experimental unit, and initial BW served as the blocking factor. The statistical model included dietary treatment as the fixed effect and block as the random effect. Least squares means were separated using the LSMEANS statement with the PDIFF option in PROC MIXED. The frequency of diarrhea was analyzed using the chi-square test. Statistical significance was declared at *P *< 0.05, and trends were noted when 0.05 < *P *≤ 0.10. Differences with *P *< 0.05 are indicated in tables, while tendencies are reported only in the text.

The metabolomics data were analyzed using various modules of the web-based platform MetaboAnalyst 6.0 (https://www.metaboanalyst.ca) (Pang et al., 2024). Peaks with detection rates below 30% were excluded, and the remaining data were normalized by logarithmic transformation and auto-scaling. Mass univariate analysis was conducted using a volcano plot, which integrates fold-change (FC) analysis with t-tests. Statistical thresholds were set at *P *< 0.10 and |FC| ≥ 2.0. Metabolites that met these criteria were subsequently subjected to pathway analysis and metabolite set enrichment analysis. Pathways were considered significantly impacted when they exhibited both a *P *< 0.05 and an impact value > 0.1.

## Results

### Growth performance and diarrhea score

Initial BW on day 0 did not differ among dietary treatment groups (Table 2). Pigs supplemented with NEO tended to have greater BW on days 7 (*P *= 0.083) and 14 (*P *= 0.089) compared to pigs fed the CON diet. Furthermore, pigs fed CBX had greater (*P *< 0.05) BW on days 7 and 14 compared to CON. NEO supplementation increased ADG and ADFI from day 0 to 7 (*P *< 0.05), as well as ADG *(P *= 0.053) and ADFI *(P *< 0.05) during phase 1. Similarly, pigs fed SCL showed a tendency for greater (*P *= 0.093) ADG from day 0 to 7 and had higher (*P *< 0.05) ADFI from day 0 to 7, 7 to 14, 14 to 21, and throughout phase 1 compared to CON. However, pigs fed SCL exhibited reduce (*P *< 0.05) ADFI from day 21 to 28 compared to the CON group. CBX supplementation improved *(P *< 0.05) ADG and ADFI from day 0 to 7, tended to improve *(P *= 0.071) ADG from day 7 to 14, and enhanced both (*P *< 0.05) ADG and ADFI during phase 1 compared to CON. Additionally, pigs supplemented with CBX exhibited reduced *(P *< 0.05) ADFI from day 14 to 21, 21 to 28, throughout phase 2, and overall experiment period compared to CON. G:F did not differ among dietary treatments throughout the experimental period.

**Table 2.** Growth performance of weaned pigs fed diets supplemented with non-nutritive sweeteners or antibiotic

Item	CON	SCL	NEO	CBX		SEM	*P*-value
**BW, kg**							
** Day 0**	6.21	6.19	6.20		6.20	0.19	0.795
** Day 7**	6.53^b^	6.59^a,b^	6.62^a,b^		6.63^a^	0.21	0.183
** Day 14**	7.96^b^	8.08^a,b^	8.17^a,b^		8.23^a^	0.25	0.125
** Day 21**	11.04	11.17	11.37		11.15	0.44	0.555
** Day 28**	14.93	15.00	15.47		15.03	0.55	0.380
**ADG, g/d**							
** Day 0 to 7**	45^b^	57^a,b^	61^a^		63^a^	5.25	0.060
** Day 7 to 14**	205	213	222		229	10.44	0.295
** Phase 1**	125^b^	135^a,b^	141^a,b^		146^a^	6.73	0.071
** Day 14 to 21**	431	439	455		417	38.41	0.378
** Day 21 to 28**	554	548	583		554	23.58	0.569
** Phase 2**	493	493	520		486	26.33	0.276
** Overall**	311	315	331		316	15.53	0.370
**ADFI, g/d**							
** Day 0 to 7**	121^b^	134^a^	136^a^		135^a^	6.13	<0.01
** Day 7 to 14**	262^b^	277^a^	265^b^		268^a,b^	8.55	0.030
** Phase 1**	192^b^	207^a^	200^a^		201^a^	7.02	<0.01
** Day 14 to 21**	579^b^	604^a^	579^b^		548^c^	26.59	<0.01
** Day 21 to 28**	863^a^	821^b^	860^a^		795^b^	23.22	<0.01
** Phase 2**	758^a^	744^a^	757^a^		706^b^	30.37	<0.01
** Overall**	449^a^	443^a,b^	453^a^		431^b^	16.76	0.017
**G:F**							
** Day 0 to 7**	0.37	0.41	0.45		0.46	0.04	0.285
** Day 7 to 14**	0.78	0.77	0.84		0.85	0.03	0.279
** Phase 1**	0.65	0.66	0.71		0.72	0.03	0.216
** Day 14 to 21**	0.74	0.72	0.77		0.75	0.04	0.454
** Day 21 to 28**	0.64	0.68	0.68		0.70	0.03	0.261
** Phase 2**	0.65	0.67	0.69		0.69	0.02	0.354
** Overall**	0.69	0.72	0.73		0.73	0.02	0.328

a,b Within a row, means without a common superscript differ (*P *< 0.05). ADFI, average daily feed intake; ADG, average daily gain; BW, body weight; CBX, CON + 50 mg/kg carbadox; CON, the nursery basal diet; Control; G:F, gain:feed; NEO, CON + 30 mg/kg neotame; Overall= weaning day (day 0) to day 28 of experiment; Phase 1= weaning day (day 0) to day 14 of experiment; Phase 2= day 14 to 28 of experiment; SCL, CON + 150 mg/kg sucralose.

Pigs fed NEO had lower (*P *< 0.05) daily diarrhea score on days 12, 21, and 23, while pigs fed SCL showed reduced (*P *< 0.05) scores on day 15 compared to CON (Supplementary Figure S1). NEO supplementation reduced (*P *< 0.05) the frequency of diarrhea during phase 1 and the entire experimental period compared to CON (Table 3). SCL and CBX groups also tended to reduce (*P = *0.059 and 0.051, respectively) the frequency of diarrhea during phase 1 and over the entire study compared to CON.

**Table 3.** Frequency of diarrhea of weaned pigs fed diets supplemented with non-nutritive sweeteners or antibiotic

Item	CON	SCL	NEO	CBX
**Frequency of diarrhea, >3**				
** Phase 1**	45.51^a^	38.46^a,b^	33.97^b^	36.54^a,b^
** Phase 2**	7.74	5.36	4.17	5.95
** Overall**	25.93^a^	21.3^a,b^	18.52^b^	20.68^a,b^

a,b Within a row, means without a common superscript differ (*P *< 0.05). CBX, CON + 50 mg/kg carbadox; CON, the nursery basal diet; Control; Frequency, number of pen days with fecal score ≥ 3; NEO, CON + 30 mg/kg neotame; Overall= weaning day (day 0) to day 28 of experiment; Phase 1= weaning day (day 0) to day 14 of experiment; Phase 2= day 14 to day 28 of experiment; SCL, CON + 150 mg/kg sucralose.

### Systemic immunity

No differences were observed in serum concentrations of TNF-α, C-reactive protein or haptoglobin, among dietary treatments on days 0, 3, and 14 (Table 4). On day 28, pigs supplemented with NEO had higher (*P *< 0.05) serum TNF-α concentrations compared to the CON group.

**Table 4.** Serum tumor necrosis factor-alpha and acute-phase proteins of weaned pigs fed diets supplemented with non-nutritive sweeteners or antibiotic

Item	CON	SCL	NEO	CBX	SEM	*P*-value
**Day 0 (weaning day)**						
** TNF-α, pg/mL**	76.11	67.31	83.06	79.10	5.51	0.267
** C-reactive protein ng/mL**	21.87	14.57	13.75	11.43	5.69	0.646
** Haptoglobin, ng/mL**	8.44	9.79	7.64	9.04	4.55	0.995
**Day 3 PW**						
** TNF-α, pg/mL**	78.39	106.51	119.55	92.91	19.41	0.276
** C-reactive protein, ng/mL**	21.52	11.34	20.02	25.10	6.73	0.427
** Haptoglobin, ng/mL**	14.61	17.61	12.91	14.55	8.95	0.987
**Day 7 PW**						
** TNF-α, pg/mL**	112.75	131.22	111.12	108.47	12.53	0.567
** C-reactive protein, ng/mL**	54.13	54.13	86.57	79.45	18.24	0.416
** Haptoglobin, ng/mL**	95.92	38.22	38.09	66.16	35.23	0.469
**Day 14 PW**						
** TNF-α, pg/mL**	116.75	127.58	115.96	106.61	13.59	0.568
** C-reactive protein, ng/mL**	62.32	54.63	51.75	68.01	12.85	0.810
** Haptoglobin, ng/mL**	79.76	96.58	97.05	87.34	32.00	0.981
**Day 28 PW**						
** TNF-α, pg/mL**	96.90^b^	109.44^b^	178.31^a^	124.36^b^	24.19	0.028
** C-reactive protein, ng/mL**	126.95	108.11	160.38	98.35	26.41	0.297
** Haptoglobin, ng/mL**	41.51	38.56	22.89	20.56	15.46	0.586

a,b Within a row, means without a common superscript differ (*P *< 0.05). CBX, CON + 50 mg/kg carbadox; CON, the nursery basal diet; Control; NEO, CON + 30 mg/kg neotame; PW, post-weaning; SCL, CON + 150 mg/kg sucralose.

### Serum metabolites

A total of 731 metabolites (248 identified and 483 unidentified) were detected in serum samples. On day 14 post-weaning, 9 to 12 metabolites were altered in each CON comparison. Compared with SCL, CON piglets showed higher montanic acid, tocopherol acetate, and deoxycholic acid, but lower indoxyl sulfate and spermidine (Supplementary Table S2). In CON vs. NEO and CON vs. NNS, several amino acid derivatives (cystine, spermidine, N-acetylaspartic acid, N-acetylornithine) and nucleosides (thymidine, adenine) were consistently reduced, whereas lipids such as montanic acid and tocopherol acetate were elevated. In CON vs. CBX, deoxycholic acid was increased, while inosine and lignoceric acid were reduced. Among non-control groups, only a few differed, including pinitol, tocopherol acetate, and cis-10-heptadecenoic acid.

On day 28 of post-weaning, more pronounced changes were observed. In CON vs. SCL, 12 metabolites differed, with increases in nucleosides (2-deoxyadenosine, thymidine, adenine), sterols (stigmasterol), and monoacylglycerols (2-monoolein), and a reduction in octadecanol. In CON vs. NEO and CON vs. NNS, differences again involved nucleosides (adenine, xanthine) and amino acid derivatives (2-ketoadipic acid, N-acetyl-D-mannosamine), alongside elevated monoacylglycerols. In CON vs. CBX, 2-monoolein was increased, while p-tolyl glucuronide and 1-monoolein were reduced. Other treatment contrasts showed modest effects, primarily involving sterols, sugars, and amino acid derivatives.

**Figure 1.**
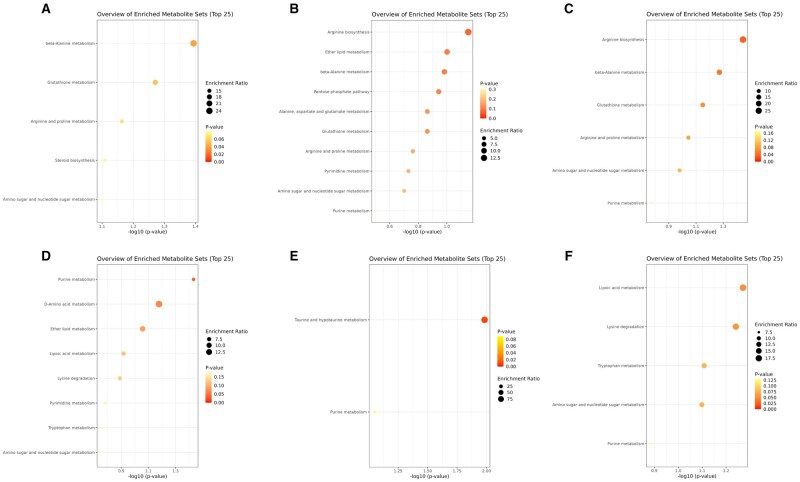
Metabolite set enrichment analysis (MSEA) of serum metabolites at day 14 (A–C) and day 28 (D–F) post-weaning. Bubble plots display the top enriched metabolite sets based on volcano plot–filtered metabolites for each pairwise comparison. The x-axis represents statistical significance (−log10 *P*-value), bubble size reflects enrichment ratio (proportion of pathway metabolites represented), and bubble color indicates *P*-value (red = lower *P*). (A) CON vs. SCL, (B) CON vs. NEO, (C) CON vs. NNS, (D) CON vs. SCL, (E) CON vs. NEO, and (F) CON vs. NNS. CBX, CON + 50 mg/kg carbadox; CON, the nursery basal diet; Control; NEO, CON + 30 mg/kg neotame; NNS, Non-nutritive sweeteners; combined metabolites of SCL and NEO; SCL, CON + 150 mg/kg sucralose.

Metabolite set enrichment analysis was performed on volcano plot-filtered metabolites to identify biological processes associated with dietary treatments (Figure 1). On day 14 post-weaning, pigs in SCL group exhibited enrichment in β-alanine metabolism and glutathione metabolism compared with CON. The CON vs. NEO contrast showed trends across several nutrient metabolic pathways, while the CON vs. NNS comparison highlighted enrichment in amino acid metabolism and amino sugar/nucleotide sugar metabolism. Comparisons among non-control groups (e.g. SCL vs. CBX, NEO vs. CBX, NNS vs. CBX) revealed fewer enrichment signals, generally limited to single amino acid–related pathways (data not shown).

On day 28 post-weaning, CON vs. SCL comparisons showed enrichment in purine metabolism, D-amino acid metabolism, and ether lipid metabolism. In CON vs. NEO, taurine and hypotaurine metabolism was the most affected, whereas CON vs. NNS revealed enrichment in lipoic acid metabolism, lysine degradation, tryptophan metabolism, and amino sugar/nucleotide sugar metabolism. Other treatment contrasts demonstrated only nominal enrichment in carbohydrate- and amino acid–related pathways, typically represented by a small number of metabolites (data not shown).

### Intestinal morphology

On day 14 post-weaning, SCL supplementation elevated (*P *< 0.05) the villi-height-to-crypt depth ratio and reduced (*P *< 0.05) crypt depth compared to the CON group (Table 5). CBX supplementation increased (*P *< 0.05) jejunal villus width on day 14 compared to SCL and NEO. In the ileum, CBX increased (*P *< 0.05) villi height and villi height-to-crypt depth ratio, while also reducing (*P *< 0.05 crypt depth) compared to CON on day 14. On day 28, pigs fed SCL showed increased (*P *= 0.063) villus width in the jejunum, but a reduced (*P *< 0.05) villi height-to-crypt depth ratio compared to CON. Also on day 28, pigs supplemented with CBX had greater (*P *< 0.05) jejunal villus width than those in the CON group. NEO supplementation had no significant effect on small intestinal morphology compared to the CON group.

**Table 5.** Intestinal morphology of weaned pigs fed diets supplemented with non-nutritive sweeteners or antibiotic

Item	CON	SCL	NEO	CBX	SEM	*P*-value
**Day 14 PW**						
**Duodenum**						
** Villi height, μm**	376	376	375	375	15.27	0.999
** Crypt depth, μm**	426	438	414	409	13.89	0.471
** Villi height: Crypt depth**	0.88	0.83	0.92	0.93	0.04	0.273
** Villi width, μm**	152	149	155	146	5.88	0.586
** Villi area, mm** ^2^	0.052	0.053	0.055	0.052	0.004	0.957
**Jejunum**						
** Villi height, μm**	336	357	356	358	16.42	0.737
** Crypt depth, μm**	325	323	317	305	10.58	0.511
** Villi height: Crypt depth**	1.05	1.12	1.10	1.18	0.05	0.387
** Villi width, μm**	122^ab^	120^ab^	118^b^	127^a^	4.32	0.117
** Villi area, mm** ^2^	0.039	0.040	0.040	0.040	0.002	0.925
**Ileum**						
** Villi height, μm**	281^b^	304^ab^	285^b^	330^a^	14.01	0.101
** Crypt depth, μm**	302^a^	267^b^	283^ab^	269^b^	10.70	0.103
** Villi height: Crypt depth**	0.90^c^	1.15^ab^	1.02^bc^	1.25^a^	0.06	<0.01
** Villi width, μm**	121	122	123	127	4.34	0.717
** Villi area, mm** ^2^	0.033	0.034	0.033	0.039	0.002	0.257
**Day 28 PW**						
**Duodenum**						
** Villi height, μm**	477	477	489	478	20.94	0.883
** Crypt depth, μm**	503	507	512	491	19.93	0.725
** Villi height: Crypt depth**	0.98	0.97	0.97	1.00	0.07	0.889
** Villi width, μm**	184	182	179	184	3.97	0.739
** Villi area, mm** ^2^	0.089	0.082	0.082	0.084	0.005	0.584
**Jejunum**						
** Villi height, μm**	437	409	425	443	16.25	0.397
** Crypt depth, μm**	352	383	379	358	14.10	0.258
** Villi height: Crypt depth**	1.27^a^	1.07^b^	1.14^ab^	1.19^ab^	0.07	0.184
** Villi width, μm**	138^b^	148^ab^	140^b^	155^a^	4.70	<0.01
** Villi area, mm** ^2^	0.056	0.061	0.056	0.063	0.003	0.274
**Ileum**						
** Villi height, μm**	406	401	416	389	17.15	0.645
** Crypt depth, μm**	327	341	339	326	13.79	0.732
** Villi height: Crypt depth**	1.25	1.20	1.25	1.24	0.08	0.934
** Villi width, μm**	148	144	147	139	3.35	0.273
** Villi area, mm**2	0.057	0.056	0.058	0.051	0.003	0.328

a,b Within a row, means without a common superscript differ (*P *< 0.05). CBX, CON + 50 mg/kg carbadox; CON, the nursery basal diet; Control; NEO, CON + 30 mg/kg neotame; PW, post-weaning; SCL, CON + 150 mg/kg sucralose.

### Intestinal barrier and innate immunity

**Figure 2.**
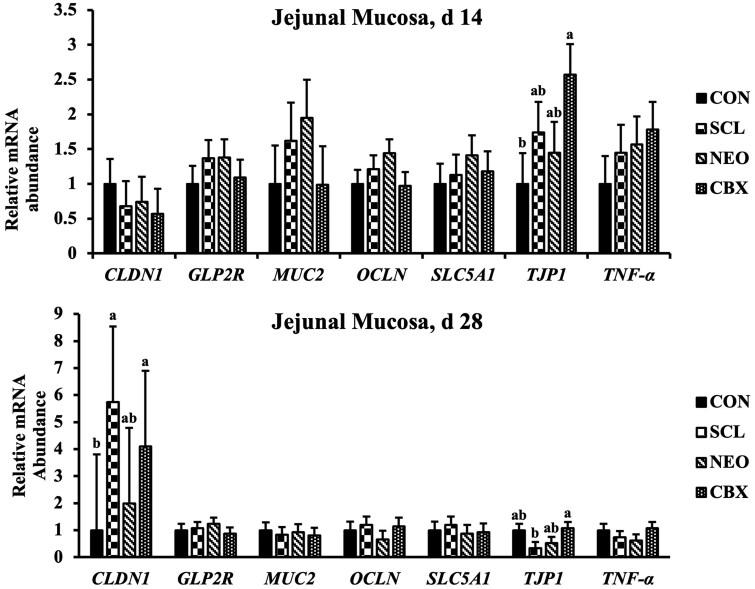
Gene expression profiles in jejunal mucosa of weaned pigs fed diets supplemented with non-nutritive sweeteners. a, b Means without a common subscript differ (*P *< 0.05). Each least squares mean represents six observations. CBX, 50 mg/kg Carbadox; CON, Control; *GLP2R*, Glucagon-like Peptide 2 Receptor; *MUC2 *= Mucin-2; NEO, 30 mg/kg Neotame; SCL, 150 mg/kg Sucralose; *SLC5A1 *= Sodium/Glucose Cotransporter 1; *TJP1 *= Tight Junction Protein 1; *TNFα*, Tumer Necrosis Factor-alpha.

**Figure 3.**
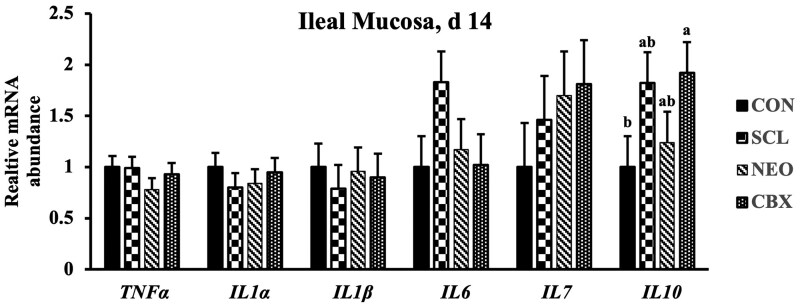
Gene expression profiles in ileal mucosa of weaned pigs fed diets supplemented with non-nutritive sweeteners or antibiotic. a, b Means without a common subscript differ (*P *< 0.05). Each least squares mean represents 6 observations. CBX, 50 mg/kg Carbadox; CON, Control; *IL10*, Interlukin-10.; *IL1b*, Interluekin-1beta; *IL1α*, Interlukin-1alpha; *IL6*, Interluekin-6, *IL7*, Interluekin-7; NEO, 30 mg/kg Neotame; SCL, 150 mg/kg Sucralose; *TNFα*, Tumer Necrosis Factor-alpha.

CBX supplementation increased (*P *< 0.05) mRNA expression of *TJP1* in the jejunal mucosa on day 14 compared to the CON (Figures 2 and 3). On day 28, SCL supplementation downregulated (*P *< 0.05) mRNA expression of *TJP1* in the jejunal mucosa compared to the CBX group. Both SCL and CBX increased (*P *< 0.05) *CLDN* expression in the jejunal mucosa on day 28 compared to CON. No dietary treatment differences were observed in mRNA expression of *GLP2R, MUC2, OCLN, SLC5A1*, and *TNFα* in the jejunal mucosa on day 14 and 28. SCL tended to increase (*P *= 0.058) the expression of IL-10 on day 14 compared to CON. CBX supplementation increased (*P *< 0.05) *IL10* expression in the ileal mucosa on day 14 compared to CON. No differences were observed in the mRNA expression of *TNFα, IL1α, IL1ß, IL6*, and *IL7* in the ileal mucosa among dietary treatments on day 14 and 28.

## Discussion

The present study evaluated the potential of two NNS, sucralose and neotame, as dietary interventions to improve growth performance, reduce diarrhea incidence, intestinal development, and systemic response in weaned pigs. Additionally, the study evaluated whether these sweeteners could provide benefits comparable to the in-feed antibiotic carbadox, thereby offering a viable nutritional intervention to alleviate post-weaning stress in antibiotic-restricted swine production systems.

In the present study, both SCL and NEO supplementation demonstrated positive effects on growth performance during the post-weaning period. NEO supplementation, in particular, improved growth performance parameters during the first 2 wk post-weaning. These findings are consistent with Zhu et al. (2016), who reported that 30 mg/kg dietary neotame increased feed intake and diet preference during the initial 10 d post-weaning, with both linear and quadratic increases observed in ADFI and ADG as inclusion levels rose. Similarly, Lee et al. (2019) observed that 0.02% dietary neotame enhanced feed preference over a 4-wk post-weaning period, supporting the role of neotame in promoting early feed intake and growth in the present study. These improvements may result from increased dietary palatability due to the sweetening properties of neotame (Nofre, 2000). In parallel, SCL supplementation in the current study improved both ADG and ADFI, specifically within the first 3 wk post-weaning. This aligns with findings by Zhang et al. (2020), who demonstrated that 150 mg/kg dietary sucralose significantly enhanced ADG and ADFI over the first 4 wk post-weaning. Mechanistic insights from Daly et al. (2021) further observed that sucralose activates sweet taste receptors T1R2 and T1R3 in the gut. Activation of these receptors by neotame or sucralose likely contributed to enhanced sensory appeal of the diet, potentially stimulating earlier and more frequent feeding behavior (De Araujo, 2011). Compared to CBX, NEO elicited comparable improvements in BW gain and ADFI during the early post-weaning phase and maintained positive effects on ADG throughout the current study. Likewise, SCL resulted in a consistent increase in ADFI across the 4-wk period. Although their mechanisms differ, NNS likely exert effects through enhanced palatability and sensory stimulation, while CBX through antimicrobial activity (Chen et al., 2020). Despite these differing mechanisms of action, the comparable improvements in growth performance observed in the present study suggest that SCL and NEO are effective nutritional interventions to support early post-weaning growth in pigs.

In addition to improving growth performance, both SCL and NEO supplementation reduced daily diarrhea scores and the frequency of post-weaning diarrhea, with NEO showing the most consistent effects throughout the experimental period. This reduction may reflect improvements in intestinal morphology and tight junction integrity, which together enhance nutrient absorption while limiting pathogen translocation and immune activation (Upadhaya and Kim, 2021). Consistent feed intake likely also contributed, as stable intake supports enzymatic activity and small intestinal function, whereas irregular or insufficient intake can disrupt digestion, promote villus atrophy, increase mucosal permeability, and trigger inflammation, ultimately leading to diarrhea (Moeser, Pohl, and Rajput, 2017). A similar relationship was observed by Sterk et al. (2008), who reported that a blend of NNS improved fecal consistency while maintaining stable feed intake, consistent with the steady intake patterns seen in pigs fed NEO and SCL during the critical post-weaning transition in the present study. Another potential mechanism involves modulation of the gut microbiota, as reduced diarrhea has been associated with a decrease in pathogenic bacteria and an increase in beneficial taxa that promote epithelial integrity and immune tolerance (Tang et al., 2022). Liu et al. (2022) found that a stevia-derived NNS decreased diarrhea while inducing favorable microbial shifts, and Lee et al. (2019) reported that neotame and a neotame + saccharin blend increased fecal lactobacillus abundance. While the current study did not assess microbial composition, these findings suggest that NNS supplementation may reduce post-weaning diarrhea through microbiota modulation, warranting further investigation.

Serum inflammatory markers remained stable during the experimental period, indicating limited systemic immune activation across dietary treatments. The transient trend observed in haptoglobin suggests a potential anti-inflammatory effect of SCL and NEO shortly after weaning, whereas the late increase in TNF-α with NEO supplementation may indicate a delayed or context-dependent immunological response. The discrepancy between this isolated inflammatory response and the generally favorable outcomes in growth performance and diarrhea warrants further investigation. Systemic immune responses to NNS, particularly sucralose and neotame, remain poorly characterized in swine. Evidence from rodent studies suggests that sucralose may influence systemic inflammation through gut microbial alterations. For instance, Bian et al. (2017) observed shifts in microbiota composition associated with inflammatory states, including increased *Ruminococcus* spp., a genus associated with gastrointestinal diseases, such as Crohn’s disease. In contrast, comparable data for neotame are limited, and further research is needed to determine whether similar mechanisms are at play. Other NNS, such as stevia, have demonstrated immunomodulatory potential in swine models. Stevia supplementation has been associated with enhanced antioxidant enzyme activities and reduced oxidative stress markers like CAT, SOD or MDA, suggesting potential benefits in controlling systemic inflammation (Liu et al., 2022). These findings highlight the importance of evaluating both oxidative stress pathways and microbial composition in future studies to better understand how NNS may influence immune function during the post-weaning period.

SCL supplementation supported early post-weaning intestinal development in the current study. These effects were most pronounced in the initial post-weaning phase but appeared less consistent by day 28, suggesting a transient impact. These morphological improvements indicate enhanced absorptive surface area and reduced epithelial turnover, both of which support more efficient nutrient absorption and contribute to intestinal stability (Tang et al., 2022). Such structural adaptions are frequently linked to stronger intestinal barrier function, potentially reducing pathogen translocation and promoting intestinal function and mucosal defense during the vulnerable post-weaning period (Tang et al., 2022). Although SCL shows positive effects on intestinal development, the broader literature on NNS and gut morphology remains limited and inconsistent, highlighting the need for further work to clarify their specific impact and underlying mechanisms. In contrast, NEO did not significantly alter intestinal morphology, suggesting that its benefits may be mediated through alternative mechanisms, particularly those involving gut microbiota. For example, Liu et al. (2022) reported that stevia residue extract supplementation did not alter intestinal morphology in weaned pigs but significantly increased beneficial microbial populations, including *Roseburia*, *Prevotella*, and *Akkermansia*, which has shown the ability to reduce intestinal inflammation. This highlights a potential mechanism whereby NEO may confer benefits through gut microbiota rather than direct structural changes to the intestine. Conversely, Chi et al. (2018) reported that neotame supplementation disrupted the mouse gut microbiome by reducing *Firmicutes*, important short-chain fatty acid producers (Meehan and Beiko, 2014), while increasing *Bacteroides*, which ferment carbohydrates but may also elevate lipopolysaccharide (LPS) production (Johnson et al., 2017). Further metabolomic analysis revealed reduced nutrient-related metabolites and increased fecal lipid excretion, suggesting impaired nutrient absorption and utilization, which may underlie the reduced BW gain observed in neotame-treated mice (Chi et al., 2018). Taken together with the present findings, where NEO improved growth performance and reduced post-weaning diarrhea in pigs, these results suggest that neotame’s interactions with the gut microbiome and host metabolism are complex and may be species-specific. This warrants the need for further research into microbial and metabolic pathways to clarify how NEO contributes to growth and reduced post-weaning diarrhea in pigs.

SCL supplementation increased *CLDN1* expression in the jejunal mucosa, encoding a barrier-forming claudin that is critical for epithelial sealing capacity (Garcia-Hernandez et al., 2017). This upregulation suggests enhanced tight junction integrity, which may contribute to enhanced gut barrier function during the post-weaning period. In terms of mucosal immune function, pro-inflammatory cytokines remained unaffected in both the jejunal and ileal mucosa, indicating that neither SCL nor NEO triggered an inflammatory or immunomodulatory transcriptional response. In contrast, Li et al. (2020) found that liquid sucralose supplementation increased colonic pro-inflammatory markers in mice, elevating cytokines, such as *TNF-α* and activating toll-like receptor-4 (*TLR4)*. In a colorectal cancer model, sucralose further increased *TNF-α* and *IL1β* while reducing anti-inflammatory markers, including *IL10* and TNF receptor-associated factor 6 *(TRAF6)*. These findings suggest that sucralose may promote gut inflammation under certain conditions, and responses may be species-dependent, as the current study observed no changes in mucosal cytokine expression in pigs. Together, these findings indicate that the tight junction improvements observed with SCL occurred independently of mucosal immune activation, suggesting a primarily structural, rather than inflammatory modulation of gut barrier function.

Untargeted serum metabolomics provided insight into systemic metabolic adaptations to dietary treatments. Early post-weaning (day 14), SCL supplementation enriched metabolites involved in antioxidant and amino acid metabolism, consistent with the observed improvements in villus architecture and tight junction expression. For instance, changes in β-alanine and glutathione metabolism suggest enhanced redox balance, which may contribute to improved barrier integrity and reduced diarrhea (Bian et al., 2017). Conversely, NEO supplementation was associated with shifts in amino acid-derived metabolites, supporting the hypothesis that its benefits may be mediated through microbial and metabolic regulation rather than direct effects on intestinal morphology. Reductions in polyamine- and sulfur-containing amino acid metabolites, coupled with altered nucleotide abundance, indicate changes in cellular turnover and host redox balance (­Grimble and Grimble, 1998; Rosa et al., 2020). Concurrent increases in fatty acids and carbohydrate intermediates point toward broader adjustments in energy metabolism. These coordinated shifts, together with enrichment of arginine biosynthesis, ether lipid metabolism, and glutathione metabolism, suggest that NEO may exert its effects by modulating pathways linked to oxidative stress defense, nitrogen utilization, and lipid remodeling rather than through structural impacts on the intestine (Smith et al., 2018; Zhang and Schroeder, 2025). By day 28, increases in nucleosides and monoacylglycerols in CON-fed pigs compared with supplemented groups highlighted weaning-associated metabolic stress. In contrast, enrichment of purine, taurine/hypotaurine, and ether lipid metabolism in SCL- and NEO-supplemented pigs suggests that dietary sweeteners may mitigate excessive energy turnover and oxidative stress during adaptation (Wang et al., 2020). The repeated identification of amino acid-related pathways across comparisons further indicates that NNS modulate host metabolism in ways that complement their beneficial effects on growth performance and diarrhea reduction (Neis, Dejong, and Rensen, 2015). Consistent trends across comparisons strengthen the interpretation that NNS influence systemic metabolism at key nodes involving amino acid catabolism, antioxidant defense, and lipid remodeling. Collectively, these findings suggest that sucralose and neotame enhance the weaned pig resilience not only through improved feed intake and intestinal health but also by modulating circulating metabolites linked to energy balance and oxidative stress. Future integration of microbiome analyses with targeted metabolomics will be critical to confirm causal mechanisms underlying these metabolic adaptations.

## Conclusion

In summary, dietary supplementation with SCL or NEO improved growth performance and reduced post-weaning diarrhea in pigs, likely through enhanced feed palatability and improved intestinal health during the critical early-weaning transition. SCL enhanced intestinal morphology and altered tight junction protein expression, suggesting a potential strengthening of gut barrier integrity. In contrast, the benefits of NEO appeared independent of structural changes in the intestine and may instead be mediated through gut microbiota modulation. Although systemic inflammatory markers remained largely unchanged, the observed subtle effects on mucosal immune gene expression highlight the need for further investigation into localized versus systemic immune modulation. In addition, untargeted serum metabolomics revealed that both SCL and NEO supplementation modulated amino acid, antioxidant, and lipid-related pathways, indicating systemic metabolic adaptations that may complement NNS’s intestinal benefits. Future research should focus on characterizing metabolomic profiles, defining gut microbial shifts, and assessing antioxidant capacity to better elucidate the mechanisms underlying these benefits. A deeper understanding of these pathways will be critical for optimizing NNS use as an alternative strategy to enhance growth and intestinal development and function in pigs under antibiotic-restricted production systems.

## Supplementary Material

skag005_Supplementary_Data

## Abbreviations:

ADFI: Average daily feed intake
ADG: Average daily gain
BW: Body weight
CAT: Catalase
cDNA: Complementary deoxyribonucleic acid
CLDN1: Claudin 1
CRP: C-reactive protein
Ct: Cycle threshold
DEPC: Diethyl pyrocarbonate
DNA: Deoxyribonucleic acid
EDTA: Ethylenediaminetetraacetic acid
ELISA: Enzyme-linked immunosorbent assay
GC/TOF–MS: Gas chromatography coupled with time-of-flight mass spectrometry
GLP2R: Glucagon-like peptide-2 receptor
IACUC: Institutional Animal Care and Use Committee
IL-10: Interleukin 10
IL-1α: Interleukin 1 alpha
IL-1βL: Interleukin 1 beta
IL-6: Interleukin 6
IL-7: Interleukin 7
MDA: Malondialdehyde
MUC2: Mucin 2
NNS: Non-nutritive sweetener
OCLN: Occludin
PBS: Phosphate-buffered saline
PCR: Polymerase chain reaction
qRT-PCR: Quantitative real-time reverse transcription polymerase chain reaction
RNA: Ribonucleic acid
RPS18: Ribosomal protein S18
SLC5A1/SGLT1: Sodium-glucose cotransporter 1
SOD: Superoxide dismutase
T1R2: Taste receptor type 1 member 2
T1R3: Taste receptor type 1 member 3
TJP1: Tight junction protein 1
TLR4: Toll-like receptor 4
TNF-α: Tumor necrosis factor alpha
TRAF6: Tumor necrosis factor receptor-associated factor 6
^ΔΔ^Ct: Delta delta cycle threshold
