# Supplementary material for: Non-nutritive sweeteners improve growth, reduce diarrhea, and modulate intestinal and systemic metabolism in weaned pigs
Source: J Anim Sci. 2026 Jan 14;104:skag005. doi: 10.1093/jas/skag005 (PMC12874886; doi:10.1093/jas/skag005)
Supplement: skag005_Supplementary_Data [file skag005_supplementary_data.zip › Supplementary Table 1.docx]

**Supplementary Table 1.** Gene specific primer sequences and PCR conditions**^1^**

| Gene^2^ | TaqMan Probe Assay | Accession^3^ Number | Forward Primer (5’ – 3’) | Reverse Primer (5’ – 3’) |
| --- | --- | --- | --- | --- |
| *RPS18* | Ss03391029_g1 | NM_213940 | TGCCTTTGCTATCACTGCGA | CTGTGGGCCCGAATCTTCTT |
| *MUC2* | Ss03377386_u1 | EU143549 | GAGAGGACTCGGTCATGCTG | AAACTCCAAGGGGCTGATGG |
| *OCLN* | Ss03377507_u1 | NM_001163647 | CAGGTGCACCCTCCAGATTG | ATGTCGTTGCTGGGTGCATA |
| *CLDN1* | Ss04329019_s1 | NM_001244539 | ATGACCCCAGTCAATGCCAG | CCCTCTCCCCACATTCGAGA |
| *TNFα-a* | Ss03391318_g1 | NM_214022 | CTGTAGGTTGCTCCCACCTG | CCAGTAGGGCGGTTACAGAC |
| *SLC5A1* | Ss03374377_m1 | NM_001164021 | GTCGCCATGGACAGTAGCAC | GATGGACAGCCCAAAGGTGA |
| *GLP2R* | Ss04322851_m1 | NM_001246266 | GAAGGGCCTACCGATACTGC | CGAAGACACAAGAGAGGGTCA |
| *TJP1* | Ss03373514_m1 | XM_021098827 | GTGGGTAACGCCATCCTCTG | GGTCGTGTGCTTCTTCCTCA |
| *IL1a* | Ss03391335_m1 | NM_214029 | GCTGAGCCTCCAGAAGAAGG | ACAACTTGGCCCAAAATGCC |
| *IL1β* | Ss03393804_m1 | NM_214055 | GCCCAATTCAGGGACCCTAC | TGTCAGCTTCGGGGTTCTTC |
| *IL6* | Ss07308316_g1 | NM_214399 | CGGATGCTTCCAATCTGGGT | TCGTTCTGTGACTGCAGCTT |
| *IL7* | Ss03394138_m1 | NM_214135 | GCAATTGCCTGAATAACGAACCT | GCTGGTGCAGTTGAACAGTG |
| *IL10* | Ss03382372_u1 | NM_214041 | CCACAAGTCCGACTCAACGA | TCTCAGGGGAGAGGTACAGC |

^1^Thermal cycling conditions were 95°C for 20 seconds, followed by 60°C for 20 seconds, and completed with 95°C for 1 second.

^2^*RPS18*: Ribosomal Protein S18; *RPL4*: Ribosomal Protein L4; *MUC2*: Mucin-2; *OCLN*: Occludin; *CLDN1*: Claudin-1; *TNFα*: Tumor Necrosis Factor-alpha; *SLC5A1*: Solute Carrier Family 5 Member 1; *GLP2R*: Glucagon Like Peptide 2 Receptor; *TJP1*: Tight Junction Protein-1; *IL1⍺*: Interleukin-1 alpha; *IL1β*: Interleukin-1 beta; *IL6*: Interleukin-6; *IL7*: Interleukin-7; *IL10*: Interleukin-10.

^3^Accession number in GenBank databas
