# Supplementary material for: Non-nutritive sweeteners improve growth, reduce diarrhea, and modulate intestinal and systemic metabolism in weaned pigs
Source: J Anim Sci. 2026 Jan 14;104:skag005. doi: 10.1093/jas/skag005 (PMC12874886; doi:10.1093/jas/skag005)
Supplement: skag005_Supplementary_Data [file skag005_supplementary_data.zip › Supplementary Table 2.docx]

**Supplementary Table 2.** Differential abundance of serum metabolites among dietary treatment groups

| **Metabolite^1^** | **Fold change** | **log2 Fold change** | ***P*-value** |
| --- | --- | --- | --- |
| CON^2^ vs. SCL^3^, d 14 PW^4^ | |  | |
| montanic acid | 5.08 | 2.34 | 0.008 |
| tocopherol acetate | 29.60 | 4.89 | 0.009 |
| indoxyl sulfate | 0.30 | -1.72 | 0.010 |
| spermidine | 0.28 | -1.82 | 0.021 |
| deoxycholic acid | 10.09 | 3.33 | 0.027 |
| 3-(4-hydroxyphenyl)propionic acid | 2.06 | 1.04 | 0.034 |
| campesterol | 2.03 | 1.02 | 0.051 |
| p-tolyl glucuronide | 0.42 | -1.25 | 0.057 |
| N-acetyl-D-mannosamine | 4.37 | 2.13 | 0.061 |
| pipecolinic acid | 0.29 | -1.77 | 0.066 |
| cis-10-heptadecenoic acid | 4.95 | 2.31 | 0.078 |
| CON vs. NEO^5^, d14 PW | | | |
| heptadecanoic acid | 2.03 | 1.02 | 0.005 |
| N-acetylaspartic acid | 0.29 | -1.80 | 0.008 |
| cystine | 0.21 | -2.23 | 0.013 |
| thymidine | 0.43 | -1.21 | 0.020 |
| octadecylglycerol | 0.31 | -1.67 | 0.056 |
| montanic acid | 2.82 | 1.49 | 0.065 |
| adenine | 0.34 | -1.54 | 0.065 |
| spermidine | 0.31 | -1.69 | 0.068 |
| N-acetylornithine | 0.16 | -2.69 | 0.076 |
| N-acetyl-D-mannosamine | 4.25 | 2.09 | 0.080 |
| 1,2,4-benzenetriol | 2.66 | 1.41 | 0.084 |
| ribose | 2.10 | 1.07 | 0.085 |
| CON vs. NNS^6^, d14 PW | | | |
| montanic acid | 3.62 | 1.86 | 0.004 |
| 3-(4-hydroxyphenyl)propionic acid | 2.00 | 1.00 | 0.008 |
| tocopherol acetate | 4.64 | 2.22 | 0.010 |
| spermidine | 0.30 | -1.75 | 0.017 |
| indoxyl sulfate | 0.33 | -1.58 | 0.018 |
| N-acetyl-D-mannosamine | 4.31 | 2.11 | 0.025 |
| adenine | 0.34 | -1.55 | 0.038 |
| cystine | 0.24 | -2.04 | 0.048 |
| N-acetylornithine | 0.26 | -1.94 | 0.084 |
| CON vs. CBX^7^, d14 PW | | | |
| deoxycholic acid | 8.74 | 3.13 | 0.040 |
| inosine | 0.34 | -1.56 | 0.066 |
| lignoceric acid | 0.42 | -1.25 | 0.088 |
| SCL vs. NEO, d14 PW | | | |
| N-acetylaspartic acid | 0.311 | -1.687 | 0.011 |
| cis-10-heptadecenoic acid | 0.075 | -3.744 | 0.019 |
| beta-glutamic acid | 0.402 | -1.313 | 0.069 |
| SCL vs. CBX, d14 PW | | | |
| pinitol | 0.474 | -1.077 | 0.002 |
| tocopherol acetate | 0.051 | -4.295 | 0.043 |
| cis-10-heptadecenoic acid | 0.172 | -2.537 | 0.054 |
| NEO vs. CBX, d14 PW | | | |
| pinitol | 0.415 | -1.268 | 0.001 |
| heptadecanoic acid | 0.494 | -1.018 | 0.009 |
| 1,2,4-benzenetriol | 0.361 | -1.469 | 0.024 |
| cadaverine | 2.117 | 1.082 | 0.090 |
| NNS vs. CBX, d14 PW | | | |
| pinitol | 0.44 | -1.17 | <0.001 |
| hypotaurine | 0.45 | -1.16 | 0.046 |
| tocopherol acetate | 0.32 | -1.62 | 0.065 |
| inosine | 0.43 | -1.22 | 0.087 |
| CON vs. SCL, d 28 PW | | | |
| 2-deoxyadenosine | 0.47 | -1.10 | <0.001 |
| 2-ketoadipic acid | 0.27 | -1.87 | 0.002 |
| thymidine | 0.46 | -1.13 | 0.004 |
| 3,6-anhydro-D-glucose | 3.42 | 1.77 | 0.007 |
| N-acetyl-D-mannosamine | 0.28 | -1.85 | 0.008 |
| adenine | 34.09 | 5.09 | 0.012 |
| propylamine | 0.43 | -1.23 | 0.012 |
| hydrocinnamic acid | 0.37 | -1.42 | 0.013 |
| octadecylglycerol | 5.85 | 2.55 | 0.022 |
| stigmasterol | 9.24 | 3.21 | 0.034 |
| 2-monoolein | 6.14 | 2.62 | 0.056 |
| octadecanol | 0.15 | -2.71 | 0.080 |
| beta-glutamic acid | 5.65 | 2.50 | 0.087 |
| CON vs. NEO, d28 PW | | | |
| p-tolyl glucuronide | 0.21 | -2.28 | 0.006 |
| xanthine | 3.04 | 1.61 | 0.016 |
| 2,8-dihydroxyquinoline | 0.45 | -1.15 | 0.022 |
| hypotaurine | 2.30 | 1.20 | 0.033 |
| 2-monoolein | 6.44 | 2.69 | 0.044 |
| CON vs. NNS, d28 PW | | | |
| 2-monoolein | 6.28 | 2.65 | 0.007 |
| 3,6-anhydro-D-glucose | 2.32 | 1.21 | 0.017 |
| p-tolyl glucuronide | 0.24 | -2.05 | 0.020 |
| 2-ketoadipic acid | 0.35 | -1.50 | 0.036 |
| N-acetyl-D-mannosamine | 0.37 | -1.42 | 0.079 |
| adenine | 2.41 | 1.27 | 0.099 |
| CON vs. CBX, d28 PW | | | |
| p-tolyl glucuronide | 0.24 | -2.07 | 0.003 |
| 2-monoolein | 7.02 | 2.81 | 0.036 |
| 1-monoolein | 0.43 | -1.21 | 0.043 |
| SCL vs. NEO, d28 PW | | | |
| hydrocinnamic acid | 6.98 | 2.80 | <0.001 |
| 2-deoxyadenosine | 2.14 | 1.10 | <0.001 |
| octadecylglycerol | 0.08 | -3.65 | 0.010 |
| xanthine | 2.77 | 1.47 | 0.016 |
| stigmasterol | 0.10 | -3.39 | 0.017 |
| N-acetylaspartic acid | 0.24 | -2.08 | 0.038 |
| succinate semialdehyde | 0.50 | -1.01 | 0.040 |
| beta-glutamic acid | 0.17 | -2.53 | 0.045 |
| N-acetyl-D-mannosamine | 2.06 | 1.04 | 0.050 |
| 1,2,4-benzenetriol | 2.01 | 1.01 | 0.080 |
| adenine | 0.04 | -4.77 | 0.091 |
| 1-methylhydantoin | 2.33 | 1.22 | 0.100 |
| SCL vs. CBX, d28 PW | | | |
| hydrocinnamic acid | 4.43 | 2.15 | 0.000 |
| 3,6-anhydro-D-glucose | 0.40 | -1.32 | 0.002 |
| aspartate | 2.48 | 1.31 | 0.004 |
| isopalmitic acid | 2.17 | 1.12 | 0.017 |
| 1-monoolein | 0.38 | -1.39 | 0.034 |
| N-acetylaspartic acid | 0.26 | -1.95 | 0.053 |
| sorbitol | 2.27 | 1.19 | 0.055 |
| octadecylglycerol | 0.23 | -2.11 | 0.058 |
| NEO vs. CBX, d28 PW | | | |
| sorbitol | 2.31 | 1.21 | 0.064 |
| galactitol | 2.13 | 1.09 | 0.071 |
| aspartate | 2.19 | 1.13 | 0.089 |

^1^Fold-change values represent the ratio of metabolite abundance between dietary treatment groups. Values greater than one indicate that metabolites were elevated in the first group compared to the second group, whereas values less than one indicate that metabolites were reduced in the first group compared to the second group. Positive log₂ fold-change values reflect higher abundance in the first group, while negative values indicate higher abundance in the second group. P-values were obtained from univariate analysis

^2^CON = the nursery basal diet; Control

^3^SCL = CON + 150 mg/kg sucralose

^4^PW = Post-weaning

^5^NNS = Non-nutritive sweeteners; combined metabolites of SCL and NEO

^6^NEO = CON + 30 mg/kg neotame

^7^CBX = CON + 50 mg/kg carbado
