# Supplementary figures and images for: Non-nutritive sweeteners improve growth, reduce diarrhea, and modulate intestinal and systemic metabolism in weaned pigs
Source: J Anim Sci. 2026 Jan 14;104:skag005. doi: 10.1093/jas/skag005 (PMC12874886; doi:10.1093/jas/skag005)

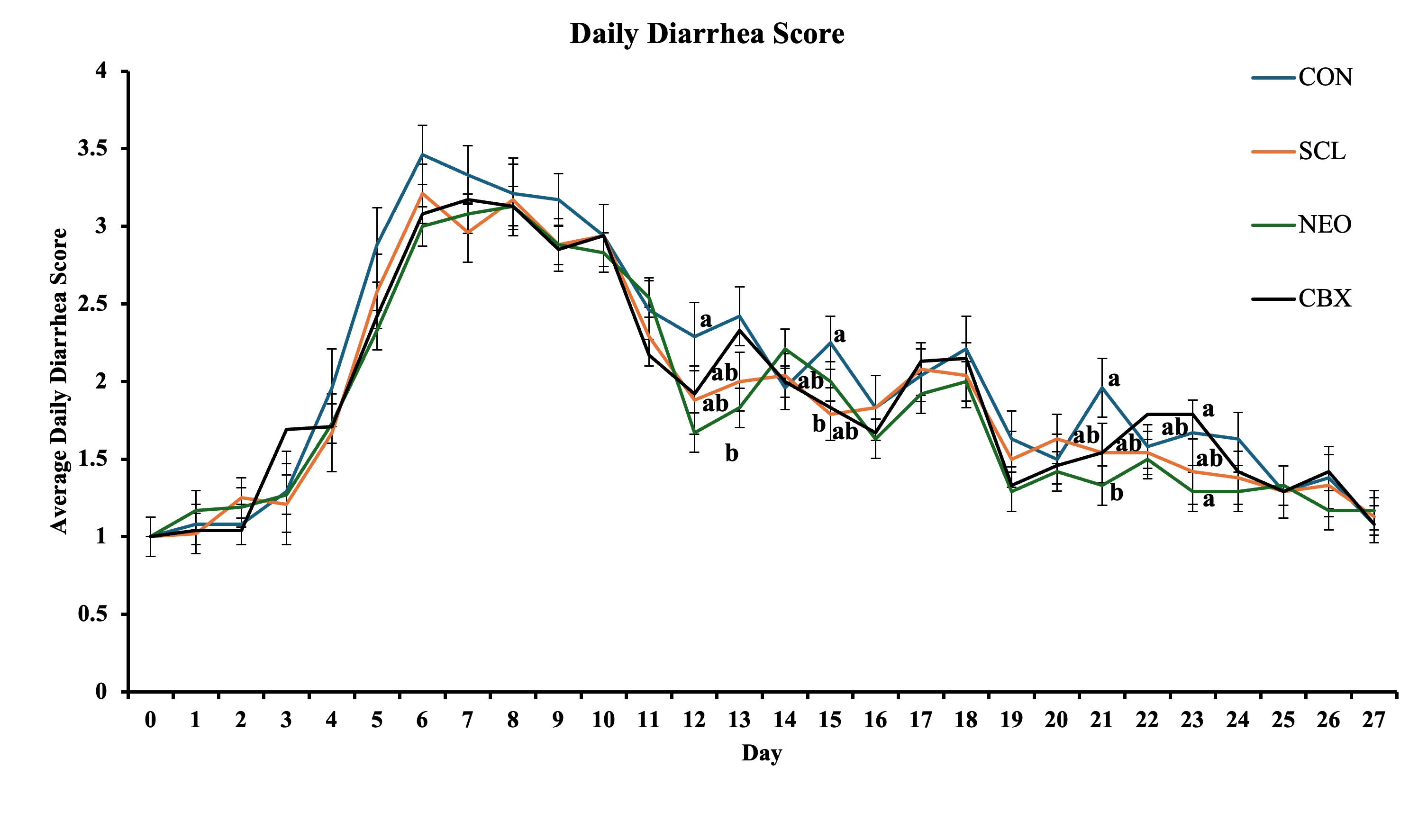

Supplement: skag005_Supplementary_Data [file skag005_supplementary_data.zip › Supplementary Figure 1.jpg]
